# Human papilloma virus infection in non-cancerous versus normal esophageal tissue samples by endoscopy

**Published:** 2015

**Authors:** Narges Mostafalou, Yousef Yahyapour, Sadegh Sedaghat, Javad Shokri Shirvani, Mahmoud HajiAhmadi, Sepideh Siadati, Shahriar Shafaei

**Affiliations:** 1 Department of Internal Medicine, Babol University of Medical Sciences, Babol, Iran; 2 Infectious Diseases & Tropical Medicine Research Center, Babol University of Medical Sciences, Babol, Iran.; 3 Department of Biostatistics, Faculty of Medicine, Babol University of Medical Sciences, Babol, Iran.; 4 Department of Pathology, Faculty of Medicine, Babol University of Medical Sciences, Babol, Iran.

**Keywords:** HPV (Human Papilloma virus), esophagitis, normal tissue; non-cancer, Real-Time PCR.

## Abstract

**Background:**

Cancers are the second most common cause of non-accidental deaths in Iran, after cardiovascular mortality. Although most cases of esophageal squamous cell carcinoma (ESCC) in the USA and western populations have been attributed to high levels of exposure to tobacco and alcohol, but in Iranian populations, other risk factors especially infectious agents have been postulated as possible causes, particularly human papillomavirus (HPV). This study aimed to determine the prevalence and the types of HPV infection in biopsy samples taken from non-cancerous esophageal lesions during upper endoscopy.

**Methods:**

A total of 80 non-cancerous esophageal samples were collected in parafinnated blocks of tissue archives in pathology. After DNA extraction, qualitative PCR (qPCR) was performed using the HPV L1 primer pairs MY09/MY11 and then genotyping was performed in HPV DNA positive by Real time PCR.

**Results:**

From 80 cases, 29 (36.3%) were qPCR positive. Using the Real-time PCR method, a total of 14 HPV genotypes were assessed. We detected HPV-11 as a dominant type in this study and we did not find any type of HPV-16 and 18 genotypes.

**Conclusion:**

In this study, HPV-II was the most common type in esophageal samples, in contrast we have found no oncogenic HPV like HPV 16 and 18 which are the most known responsible factors of ESCC in other countries.

Cancers are the most common cause of death throughout the world. It is estimated that the overall incidence of different types of cancers will have increased by 45% in developed countries by 2030. Many reports indicate that cancers are the second most common cause of non-accidental deaths in Iran, after cardiovascular events (1, 2). Carcinoma of the esophagus is a common malignancy and its mortality rate is among the highest in all cancers (3). There is a wide geographic variation in the incidence of esophagus cancer, with a difference as much as 300-fold between the areas with highest and lowest prevalence across the countries (4). Considerable alcohol use combined with smoking was found to enhance the risk of esophageal cancer in Western countries (5). In contrast, vitamin deficiency and food containing probable carcinogenic substances have been considered as important risk factors for esophageal cancer in China and other central Asian countries (6). Nonetheless, the association between esophageal cancer and these recognized risk factors is usually weak for high risk population of China, implying other etiological factors contributing (7). 

Both histological subtypes of esophageal malignancies, including squamous cell carcinoma (SCC) and adenocarcinoma (ADC) are highly lethal with a current five-year survival of less than 10% (8). In the USA and other western countries, tobacco, too much drinking alcohol, diets poor in fresh fruits and vegetables, and low socioeconomic group have been associated with esophageal SCC (7, 9). In Iran and China, alcohol and tobacco are not considered as risk factors for esophageal cancer (7, 10-14).

Human papilloma virus (HPV) is a viral agent associated with esophageal cancer thus far. HPV, especially genotypes of 16 and 18, were the major cause in uterine cervical carcinoma and has a strong association with cancers of the vulva, anus, penis, oropharynx and esophagus (15). HPV types 16, 18, 31, 33, 35, 39, 45, 51, 52, 56, 58, 59, 68, 73, and 82 are classified as high-risk (HR) oncogenic types; types 6, 11, 40, 42, 44, 54, 61, 70, 72, 81, and CP6108 are classified as low-risk (LR) types; and types 26, 53, and 66 are considered as probably oncogenic. Also, HPV types 16, 18, 31, 33, 35, 39, 45, 51, 52, 56, 58, 59 and 66 are carcinogenic to humans (Group 1). HPV 6 and HPV 11 (Group 2B) and some types of HPV genus beta are possibly carcinogenic to human (Group 2B) (16). Serologic studies as well as studies considering HPV-DNA (17) revealed inconsistent data and found the evidence of exposure to HPV in 0-67% of cases (18, 19). 

In many Iranian studies, esophageal SCC was associated with HPV 16 but not HPV 18 (20). Most infected people are asymptomatic and so HPV infection will be unrecognized because the immune system inactivates the virus. In about 90% of people, the immune system clears the HPV infection within 2 years. 

This is true for both the high-risk and low-risk HPV types. Sometimes HPV infections are not clear and results in cell changes which may predispose the infected to the development of future cancers (21). The present study aimed to assess HPV infection in samples provided during endoscopy from patients with non-cancerous lesions or normal esophagous.

## Methods

**Subjects: **A total of 80 non-cancerous esophageal samples were collected. The presenting symptoms in 48 patients was dyspepsia, 20 patients had heart burn, and 12 patients with dysphagia. 57 samples were provided from Shahid Beheshti Hospital, one of the big hospitals in Babol, a city in Mazandaran, North of Iran; 23 samples were obtained from Central Lab of Amol, a city, west of Babol. Demographic and medical information including age, gender, resident, smoking and type of lesion were obtained from the patients’ medical records. Based on pathology diagnosis, the parafinnated blocks of tissue-archive in two centers in patients with non-cancerous esophageal lesion were entered in our study. 

**DNA extraction and Real Time PCR: **The methods were described previously (16, 22). After DNA extraction, Real-time PCR was performed with the Corbett Rotor-Gene 6000 Sequence Detection System and SYBR-Green PCR master mix (Maxima® SYBR Green qPCR Master Mix (2X), Applied Fermentas, EU). The primers used in this study were general primers from MY09 and MY011 pairs (MY09: 5'-CGT CCM AAR GGA WAC TGA TC-3' and MY011: 5'-GCM CAG GGW CAT AAY AAT GG-3').

All HPV-DNA positive specimens of esophageal biopsy with non-cancerous lesion for HPV genotyping performed with high-sensitive real-time PCR (Rotor Gene 6000, Corbett Research, Australia & USA) by AmpliSense HPV HCR genotype FRT PCR Kit for High-Risk genotyping using AmpliSense HPV 6/11 FRT PCR Kit for the detection of Low-risk genotyping (Federal State Institution of Science Central Research Institute of Epidemiology, 3A Novogireevskaya Street Moscow 111123 Russia).

## Results

The patients were 42 to 90 years old and their mean±SD age was 65±12.3 years. The characteristics of patients and the frequency of HPV infection were presented in table 1. 

Using the Real-time PCR method, a total of 14 genotypes including HPV6, 11, 16, 18, 31, 33, 35, 39, 45, 51, 52, 56, 58 and 59 were analyzed. As seen in table 2, we detected HPVs-11 (in 4 cases), 33 (in one case), 56 (in 2 cases) and HPV-56 (in one case) of patient with inflammation. Also, HPVs-11, 39 and 52 were detected in only one case of patient with dysplasia. The most prevalent genotype was HPV11 (17.2%; 5.29) of the HPV positive cases (36.25%; 29.80) and the second more prevalent genotype was HPV 56 with 6.9% (2.29). Also, HPV was found in 8 inflammatory samples in 4 cases, HPV 33 and 58 in one case and HPV 56 in 2 cases). In 12 dysplastic samples, 3 genotypes of HPV including HPV-11, 39 and 52 were detected.

**Table 1:** Patient characteristics and frequency of HPV according to subgroups

	**N(%)**	**P-value**	**HPV** ^+^ **N (%)**
**Gender**			
Male Female	48 (60) 32 (40)	0.483	18 (37.5) 11 (34.3)
**Age**			
<50 50 to 65 ≥ 65	11 (13.7) 27 (33.7) 42 (52.5)	0.521	3 (27.3) 11 (40.7) 15 (35.7)
**Histopathological diagnosis**			
Inflammation Dysplasia Barrett’s esophagus Normal tissue	51 (63.7) 12 (15) 4 (5) 13 (16.2)	0.955	20 (39.2) 4 (33.3) 1 (25) 4 (30.8)
**Resident**			
Urban Rural	44 (55) 36 (45)	0.001	23 (52.3) 6 (16.7)

## Discussion

In our study, from the 80 cases with tissue diagnosis of non-malignant esophageal lesions, the prevalence of HPV-DNA in these samples with Real Time PCR was 36.3%. We reported, 20 (39.2%) HPV DNA in esophagitis samples and others were 29.6% (23), 58.8% (24) and 26% (25). Also, we reported 4 (33%) HPV DNA in dysplasia. This is less than in Mohiuddin’s 71.4% (24) and Liu’s studies about 70% (26). In our study, 1 (25%) HPV DNA in Barrets samples and other studies reported 96% (25), 27.4% (27), 1.7% (28), respectively. We reported 4 (30.8%) HPV DNA in normal samples and other researchers showed 45.7% (24), 33% (26) and 24.1% (27).

Many studies were performed in different geographic parts of Iran, from northeast of Iran (29), northwest of Iran (30), north of Iran (31) and central of Iran (20), HPV prevalence reported 49.4% (in ESCC), 37.7% (in ESCC), 12.5% (in non-cancerous samples) and 13.2% (in non-cancerous samples), respectively. 

Other studies from many countries including China (0- 71%) (17, 32, 33); India (57.8%) (24); Brazil (0.0%) (34); Italy (29.6% in esophagitis) (23) and Australia (0.0% in normal tissue samples) (35) have reported the patients with non- malignant esophageal. Therefore, in our study we found 36.3% HPV DNA in non-malignant esophageal patients that is similar to many areas of China and less than India, but greater than Brazil, Italy and Australia. Several studies reported different prevalence of HPV in normal and malignant tissue and the range of positivity was between 0–82% (17, 32).

Yahyapour et al. reported HPV in 27% of ESCC patients. They found one case of HPV-16 and one case of HPV-18. They reported the highest genotypes HPV 11 & 45. Also, they reported the etiology of HPV 16 & 18 for infection of by cancer in the North of Iran (16, 20). Therefore, many studies in the North of Iran are similar to our study and confirmed some high risk HPV genotypes (HPV-16 and 18) are not as a main risk factor for esophageal disease (16). However, Liu et al. reported HPV-16, 18, 58, 31 as high risk types and HPV-6 and 11 as low risk types in dysplasia and normal tissue samples (26). In Acevedo-Nuno’s study, the major HPV viral types detected by PCR in positive samples were strains 6 and 11, while less frequent were strains 18, 31, 33, 35, 51 and 58 (25). Many HPV genotypes including 11, 33, 39, 52, 56 and 58 were detected and HPV 11 was the dominant type in this study (17.2%; 5/29) of the HPV positive cases (36.25%; 29/80) and the second more prevalent genotype was HPV 56 with 6.9% (2/29) (table 2). In our study, we did not find HPV 16 &18 genotypes, similar to Yahyapour (16, 22), Farhadi (20) and Antonsson’s (35) studies and are different from Tahmasbi (36) and Emadian’s studies (31). In our study, we did not find HPV 16 & 18 genotypes but Tornesello in Italian patients with esophagitis detected HPV-16, 19, 20 and 25 (23). Also, Emadian of the North of Iran reported HPV- 16 and 45 in many normal tissue samples (31). In our opinion, many different methods, type of primers used, geographical region and type of specimens can affect the diversity of HPV prevalence and their types. 

In our study HPV prevalence in ≤55 years was 26.3% and ≥55 were 39.3% (p>0.05). Also, infection to HPV in man was detected 37.5% versus 34.3% in women. Also, we found HPV infection in urban patients was more than rural patient samples (P=0.001) and those results were not reported in other studies. To compare, in Qi’s study in China, HPV prevalence in ≤55 years were 25% and >55 was detected in 30.9%; also, they reported HPV infection in men about 26.5% versus 41% in women (33).

In the study of Tornesello, 51.8% of infection specimens those less than 50 years old and 11.1% older than were 60 years. Also, 70.4% of males and 29.6% of females were HPV DNA positive (23). But, Dabrowski reported that the rate of HPV infection in females was less than males (37). It seems that, if HPV infection occurs in younger age then the risk of cancer is higher in elderly age. Heterogenesity in geographic and racial prevalence is one of the most common characteristics in esophageal cancer. The highest prevalence in the world is Transki of South African, northern Iran, China, South America, and southern France. Esophageal cancer is one of the leading causes of mortality of cancer especially in developing countries (38). 

In conclusion, this study evaluates the prevalence of HPV infection in non-cancer tissue samples in Mazandaran, North of Iran for the first time in our knowledge. From the results of this study, HPV DNA prevalence in non-cancerous samples of esophagus in North of Iran in comparison to other geographic study is considerable. Some oncogenic types of HPV (HPV-16 and 18) were not found in our samples. Therefore, we suggest that there are different infections of HPV genotypes in patients with non-cancerous lesion of esophagus in the North of Iran with other parts of Iran and other countries. 

## Notes

**Funding:** This study was supported by a grant (number 923547) from Babol University Infections Diseases and Tropical Medicine Research center. Also, this article is a thesis of an internal medicine resident.

## Notes

**Conflict interest: **There was no Conflict of Interest.
